# Cell membrane asymmetries and cellular aging

**DOI:** 10.1042/BCJ20253265

**Published:** 2025-10-09

**Authors:** Valentina Salzman, Pablo S. Aguilar

**Affiliations:** 1 Instituto de Fisiología, Biología Molecular y Neurociencias (IFIBYNE), CONICET, Buenos Aires, C1428EGA, Argentina

**Keywords:** aging, asymmetric cell division, membrane, yeast

## Abstract

*Saccharomyces cerevisiae*, a widely studied unicellular eukaryotic model, multiplies and divides through an asymmetric budding process, where a mother cell produces a smaller daughter cell. Although cells age across successive cell divisions, provided the mother is not too old, each daughter cell inherits a full lifespan potential. Extensive studies in budding yeast have established a framework for understanding how asymmetric cell division contributes to this lifespan resetting. One postulate of this framework is that the capacity of mother cells to bud daughters with full replicative potential is critically dependent on membraneassociated mechanisms that enable asymmetric inheritance of aging factors. Despite the identification of numerous asymmetrically distributed proteins, an integrated catalog detailing their roles in aging has not been compiled. This review provides a comprehensive resource of asymmetrically distributed membrane proteins in yeast that have a role in replicative aging. Existing knowledge governing the establishment and maintenance of asymmetry is synthesized, and gaps in our understanding of how membrane asymmetry contributes to cellular aging are identified.

## 1. Introduction

As a powerful model organism, budding yeast research has revealed numerous pathways linked to aging across species demonstrating its value for understanding this process in other eukaryotes, including mammals [1,2]. Aging in *Saccharomyces cerevisiae* is primarily assessed using two distinct assays: replicative lifespan (RLS), which measures the number of divisions a cell can complete before arresting and entering senescence, and chronological lifespan, which measures the survival time of non-dividing cells in stationary phase [2]. RLS and asymmetry during cell division are the focus of this review.

In *S. cerevisiae,* each cell division produces a daughter that buds off from an aging mother. The replicative potential of the mother cell is limited; it produces on average 25 daughters until it dies. Despite originating from aged mother cells, daughter cells exhibit full replicative potential; i.e. only daughters of very old mothers inherit aging markers and have a reduced lifespan [3,4]. Budding yeast research supports a central role for the unequal distribution of cellular damage as a means to generate fully young cells from aging mothers and thus control their lifespan.

Yeast mother cells have the capacity to retain damaged or harmful cellular components and prevent their inheritance to the bud. These segregated components include extrachromosomal rDNA circles (ERCs), protein aggregates, vacuoles with increased pH, and mitochondria with decreased membrane potential [1]. Importantly, many of these processes involve membrane proteins, which play essential roles in controlling cellular damage asymmetry by mediating organelle transport and localization and selective anchoring of protein aggregates and ERCs, among other functions. Consequently, studying the behavior of membrane proteins during cell division can reveal fundamental aspects of cellular aging and longevity regulation.

Our understanding of the relationship between lifespan and asymmetric cell division is still limited. While a subset of analyses identifying asymmetrically distributed yeast proteins characterized their impact on aging, 40% of genes encoding asymmetrically segregating proteins lack a direct assessment of their RLS phenotype [5–9]. The goal of this review is to address this limitation by synthesizing existing knowledge on the asymmetric distribution of aging factors. We present a curated list of asymmetrically distributed membrane proteins, highlighting those that have a proven role in RLS and detailing evidence for asymmetric distribution and biological roles. Next, we describe interplays found between these membrane proteins and key aging pathways. Finally, we discuss the most relevant questions and future research directions to obtain a comprehensive picture of cell membrane asymmetry and the aging process.

## 2. Asymmetric distribution of aging factors

### 2.1 Mitochondria asymmetric segregation

Early evidence for mitochondrial decline involvement in budding yeast aging came from population-level studies reporting that old mother cells often produced petite daughters—slow-growing progeny with respiratory defects arising from mutations or complete loss of mtDNA [10]. This model was reinforced by direct observations of declining mitochondrial membrane potential in aged mothers [11]. Yet, insights from single-cell analyses using microfluidics [12] showed that only a subset of aging mothers experiences progressive mitochondrial decline. Such approaches reveal the heterogeneity within aging populations that bulk studies mask. These findings support a model in which this mitochondrial decline occurs stochastically and independently of age, challenging the idea that it is a universal hallmark of replicative aging in yeast. More recently, Li et al. [13] also found heterogeneous replicative aging subpopulations in budding yeast. They proposed a model in which aging proceeds toward distinct states, with one trajectory characterized by progressive mitochondrial decline. The combined findings of these studies indicate that once established, mitochondrial decline—whether stochastic or age-associated—leads to cell death. Mitochondria within budding yeast exhibit functional diversity, as evidenced by variations in membrane potential and oxidative state [14]. The heterogeneity arises partly from damage caused by mtDNA mutations and reactive oxygen species (ROS), which originate both inside and outside the organelle [15,16]. To counteract this damage, budding yeast employ mitochondrial quality control processes that either repair or remove severely damaged mitochondria [14]. Nevertheless, the cellular mechanisms for mitochondrial repair and regeneration become less effective with age, leading to a general decline in mitochondrial function. Moreover, age-associated changes in vacuolar pH described in section 2.4 also limit mitochondrial function [11]. As a result, activating mitochondrial quality control pathways has been shown to delay aging and increase RLS [17,18]. Daughter cells’ mitochondria contain less ROS, are more reduced, and possess a higher membrane potential than mitochondria in mother cells [14]. To reach this asymmetric distribution, fitter mitochondria are preferentially inherited by daughter cells during cell division, while lower-functioning mitochondria are retained in mother cells [19]. The role of the cytoskeleton in this process is widely studied, and readers are encouraged to consult recent reviews [14,20]. Briefly, mitochondria undergo linear, polarized movement that is mediated by actin cables through membrane-cytoskeleton interactions. This partitioning of mitochondria depends on highly regulated binding of myosin motor proteins to cargo membranes [14]. However, the way mitochondrial fitness is sensed and how the organelles become both associated with and released from myosins at appropriate times and locations is not well understood [21–23]. Nevertheless, key tether proteins responsible for immobilizing mitochondria at specific locations to maintain asymmetry have been characterized.

Two major mitochondrial retention mediators are Mmr1, which anchors fitter mitochondria in the bud tip, and Num1, which tethers mitochondria to the mother cell cortex. Mmr1 is a phospholipid-binding protein that is peripherally associated with the mitochondrial outer membrane [24]. While Mmr1 overexpression enhances mitochondrial accumulation in the bud [25], its deletion impairs bud tip anchorage of mitochondria [26] and decreases RLS [27]. By contrast, Num1 anchors lower-functioning mitochondria to the mother cell plasma membrane [14]. Num1, the core component of the MECA, is a phospholipid-binding protein that binds to both plasma membrane and mitochondrial membrane-specific lipids [28,29]. Its absence leads to increased mitochondrial motility, distribution defects [14], and decreased RLS [30].

Interestingly, retention of a small population of higher-functioning mitochondria at the distal tip of the mother cell is also necessary for mother cell fitness and RLS in budding yeast [27,31]. Mfb1 plays a key role in this process by tethering these high-performing mitochondria at the mother cell tip during cell division. Therefore, *MFB1* deletion results in a significant reduction in overall mitochondrial quality in the mother cell and a decrease in RLS [14].

Asymmetric segregation of mitochondria in mammalian stem-like cells (SLCs) has also been shown [32]. Katajisto et al. [33] reported that human mammary SLCs asymmetrically segregate aged mitochondria during cell division, daughter cells receiving fewer old mitochondria maintained stem cell traits. Outer mitochondrial membrane proteins appear to play a role in the segregation process. A high-performing mitochondrial population was found to be tethered to the cell cortex through a complex proposed to comprise mitofusin MFN1, which is required for mitochondrial fusion, and the kinase PKCζ from the PKC family [34]. Remarkably, the cortex-tethered mitochondria that were segregated into the SLCs progeny displayed increased ROS scavenging capacity. However, the mechanisms involved in mitochondrial fitness segregation within mammalian stem cells that divide asymmetrically remain largely elusive.

### 2.2 Asymmetric segregation of extrachromosomal rDNA circles

Genes encoding rDNA are organized into a repetitive array in eukaryotic genomes. Their copy number (CN) in *S. cerevisiae* commonly ranges from 100 to 250 copies per genome [35]. When an intramolecular homologous recombination event occurs at the rDNA locus, single rDNA units can be excised from the chromosome as ERCs. ERC accumulation is not limited to yeast but has also been observed in other organisms, such as amphibians, flies, and protozoa [36].

Several lines of evidence establish that ERC accumulation promotes cellular aging in *S. cerevisiae*. Notably, the artificial introduction of ERCs into a young budding yeast cell is sufficient to accelerate aging (Sinclair and Guarente 1997; Falcón and Aris 2003).

It is proposed that ERC accumulation accelerates aging by different mechanisms such as perturbation of nucleolar function, increase in genomic instability, and titration of key nuclear proteins such as Sir2. Proteins acting at the rDNA locus modulate rDNA stability, consequently influencing the formation of ERCs. For instance, Fob1 diminishes rDNA stability and thereby promotes ERC production [36]. Fob1 exerts this effect by directly binding to the replication fork barrier in the rDNA locus, stalling the replication fork and inducing DNA double-strand breaks [37]. The DNA broken ends trigger intra-chromatid recombinational repair, resulting in the release of ERCs. Conversely, the Sir2 deacetylase promotes rDNA stability and reduces ERC formation. Sir2 deacetylates histones at the rDNA, which leads to repression of E-pro transcription, a non-coding RNA promoter. This transcriptional repression, in turn, helps maintain cohesin association throughout the rDNA locus, thereby preventing recombination and ERC formation. Inactivation of Sir2 leads to increased E-pro transcription, cohesin dissociation, and the accumulation of ERCs [37]. *sir2Δ* and *fob1Δ* strains exhibit shortened and extended lifespans, respectively [38,39]. It has been reported that there is a positive correlation between the rDNA CN and RLS. The proposed mechanistic basis for this observation is that strains with a low rDNA CN (and shorter lifespan) display reduced levels of Sir2 expression, leading to a more rapid accumulation of ERCs [35]. Recent findings challenge the role of ERCs pointing to the accumulation of rDNA within linear fragments of chromosome XII as a driver of aging in budding yeast [40].

There is compelling evidence showing that during budding yeast cell division, ERCs are retained within mother cell nuclei [1]; however, the segregation mechanism is still not clear. A line of evidence suggests that retention of ERCs in the mother cell depends on their anchorage to the nuclear membrane periphery, through nuclear pore complexes (NPCs) [41]. Recently, it has been postulated that attachment of ERCs to NPCs causes NPCs to lose their nuclear basket and cytosolic subcomplex integrity. Preventing the acetylation of Nup60, one of the basket proteins displaced from ERCs-bound NPCs, restores the localization of the cytosolic complex and increases RLS [42]. This suggests that NPC cytosolic disassembly is intrinsically linked to the mechanisms by which ERCs promote aging. However, ERCs’ anchorage does not appear to damage NPCs per se but rather to activate a regulatory process that involves the compositional and functional reorganization of NPCs. Future research will be crucial to dissect the intricate relationship between ERCs anchorage, NPC dynamics, and RLS. Other studies challenge the NPC-dependent segregation of ERCs, indicating that they can freely diffuse in the nucleoplasm and that the natural geometry of budding yeast cell division, with its closed mitosis, is sufficient to limit ERC diffusion into daughters [1,43]. It remains to be determined to what extent these different models contribute to ERCs’ retention.

### 2.3 Asymmetry in damaged protein aggregates segregation

Cytosolic protein aggregates accumulate with time in several model organisms and are considered a hallmark of aging. The age-related accumulation of damaged proteins is proposed to result from both increased exposure to damaging agents (e.g. ROS [44]) and a decreased capacity of the protein quality control (PQC) system to remove damage. This system mediates the *de novo* folding of newly synthesized polypeptides, the refolding of misfolded proteins, and the degradation of proteins that cannot be refolded [45]. The PQC system is continuously at work to maintain low levels of protein damage; however, accumulation of misfolded proteins appears inevitable during aging. Misfolded proteins can accumulate into aggregates at multiple sites throughout the cytosol and on the cytosolic surface of various organelles, including the ER [46], nucleus [47], mitochondria [48], and vacuole [49]. Moreover, to cope with the accumulation of protein aggregates, cells sequester these aggregates into large cytosolic protein inclusions (PIs) at sites located next to cell membranes: the juxtanuclear quality control site (JUNQ) and the peripheral vacuole-associated insoluble protein deposit (IPOD) [50]. In addition, misfolded protein aggregates can also form a PI in the intranuclear quality control site (INQ). While PI formation is not essential for degradation, it may facilitate both refolding and degradation by concentrating chaperones and their substrates within the cell [51].

Several classes of proteins are involved in the recognition and elimination of protein aggregates. Small heat shock proteins, referred to as holdases or aggregases, bind misfolded proteins and concentrate them in stable aggregates to prevent further interactions. Co-chaperones of the J-protein family (Hsp40) are also early participants in the aggregate recognition process. Hsp70 chaperones have protein-folding capacities (foldases) and also aid in protein degradation [45]. When intracellular H₂O₂ levels rise, chaperone recruitment requires the peroxiredoxin (Prx) Tsa1, a peroxide scavenger that also works as a chaperone [52]. Proteases, such as Mca1, which is needed for aggregate removal, also participate in this process [53]. Finally, the Hsp104 chaperone is key for aggregate sequestration into PIs [54].

Multiple lines of evidence suggest an age-related decline in the cell’s ability to manage damaged proteins [50]. Deletion of major chaperones, ROS scavengers, or proteasome subunits leads to an increased load of non-native proteins and protein aggregates and reduces RLS in budding yeast [45]. Conversely, increasing the activity of PQC components, such as Tsa1 and Mca1 [52,53], mitigates the accumulation of protein aggregates in aged cells and extends RLS.

During *S. cerevisiae* mitosis, buds emerge free of protein aggregates and PIs, which are efficiently retained by the mother cell [50]. Retention has been proposed to occur through organelle and cytoskeletal attachment and by cell geometry-restricted diffusion [1,55]. Moreover, the sequestration of protein aggregates into PIs within the mother cell is suggested to be necessary for preventing the inheritance of smaller aggregates by the daughter cell [45,56]. Proteins such as Hsp104, necessary for PIs formation, are thus relevant for proper aggregate retention. Not only does *HSP104* gene deletion disrupt aggregates asymmetric partition and decrease RLS [54] but also Hsp104 protein levels positively correlate with RLS [57], likely because Hsp104 activity is needed for damaged protein sequestration into PIs [56]. The histone deacetylase Sir2 has also been implicated as an important factor for aggregate partition. The *SIR2* deletion mutant strain shows a more symmetrical distribution of carbonylated proteins [54]. Erjavec et al. demonstrated that Hsp104 overproduction improves damage retention and suppresses the reduced RLS phenotype of the *sir2* mutant. The mechanisms responsible for asymmetric segregation of protein aggregates are still largely unknown. However, a consensus indicates that an intact actin cytoskeleton and cytoskeleton remodeling proteins are required for the selective retention of aggregates [45]. Moreover, it is suggested that Sir2 affects asymmetry, possibly by regulating actin assembly [54,58]. In addition, close localization of aggregates to organelles suggests tethering to organellar membranes as a possible mechanism of segregation [45]. Evidence suggests that ER membrane-bound chaperone Hsp40 can capture deposits of aggregates and retain them in mother cells [46]. Some features of inclusion formation and damage retention that are seen in yeast are also conserved in higher eukaryotes [45]. Mammalian JUNQ inclusion bodies, which sequester cytosolic misfolded proteins, are inherited asymmetrically in certain cell lines, mirroring the behavior observed in their yeast counterparts. These JUNQ inclusions contain protein aggregates and accumulate chaperones, such as Hsp70, and active proteasomes [59]. Ogrodnik et al. further propose that the mammalian cytoskeleton, specifically vimentin intermediate filaments, provides the physical scaffold for asymmetric inheritance of JUNQ inclusions [59].

### 2.4 Vacuole acidity asymmetry

Aging in *S. cerevisiae* is characterized by a gradual increase in cytosolic pH, resulting in decreased vacuolar acidity in mother cells. Age-dependent loss of acidification impairs vacuolar function and leads to mitochondrial fragmentation and dysfunction and an increase in ROS production [11].

Unlike their mothers, daughter cells have a low vacuolar pH [60]. During cell division, a portion of the mother cell’s vacuoles is inherited by the bud through a process that requires actin and the motor protein Myo2. As a vacuole-specific cargo adaptor, the Vac17 protein bridges Myo2 and the vacuole during yeast budding [61]. Consequently, *VAC17* gene deletion leads to defects in the initiation of vacuole transport. In addition to Vac17, Vac8 is also essential for this process [62]. Vac8 is an Armadillo repeat-containing protein that resides on the vacuolar membrane and mediates diverse cellular functions [63]. Myo2 and Vac8 interact in a Vac17-dependent manner [62], suggesting that Vac17 acts as a bridge between the two proteins. The vacuole inheritance complex Myo2-Vac8-Vac17 ensures organelle transfer to the daughter cell, but there is no evidence whether this process is associated with the pH of the vacuole. Instead, it is proposed that newly inherited vacuoles in the daughter cell undergo processes that re-establish their optimal (acidic) pH. Maintaining an acidic lumen, crucial for vacuole function, is largely accomplished by the vacuolar proton-ATPase (V-ATPase) [64]. This proton pump actively transports protons out of the cytosol [65] and is localized not only on the vacuolar membrane but also on organelles of the secretory pathway, such as endosomes and the Golgi apparatus. It is proposed that the differential acidification of vacuoles between mother and daughter cells is driven by a corresponding difference in their cytosolic pHs [11,60]. Pma1, the yeast’s main plasma membrane proton-ATPase that pumps protons out of the cell, is present in mother cells but not in their newly formed daughter cells [7,8]. The mechanism underlying this asymmetry remains unclear [9,66]. Henderson et al. [60] found that the inherent asymmetry of Pma1 increases cytosolic proton availability in daughter cells and facilitates vacuole re-acidification and its consequent rejuvenation. Authors suggest that, due to the mother-enriched distribution of Pma1, both cytosolic and vacuolar pH increase in aging mother cells. Consistent with this model, artificial reduction in Pma1 activity in mother cells made the entire cell more acidic, and so did their vacuoles [60]. Conversely, daughter cells engineered to contain more Pma1 were less acidic and had less acidic vacuoles than normal.

## 3. Asymmetrically segregated membrane proteins and their interplay with aging pathways

Research on the asymmetric distribution of aging factors in budding yeast, as described in Section 2, reveals that yeast mother cells retain damaged components through processes often involving membrane proteins. Furthermore, the changes in organelle characteristics and molecule accumulation between mother and daughter cells are at least partially mirrored by membrane asymmetry. However, our understanding of the link between lifespan and asymmetric division is limited. To address this issue, an integrated catalog of asymmetrically segregated membrane proteins in budding yeast, detailing the roles of these proteins in aging, has been compiled.

### 3.1 Identifying asymmetrically segregated membrane proteins

**Figure 1:**
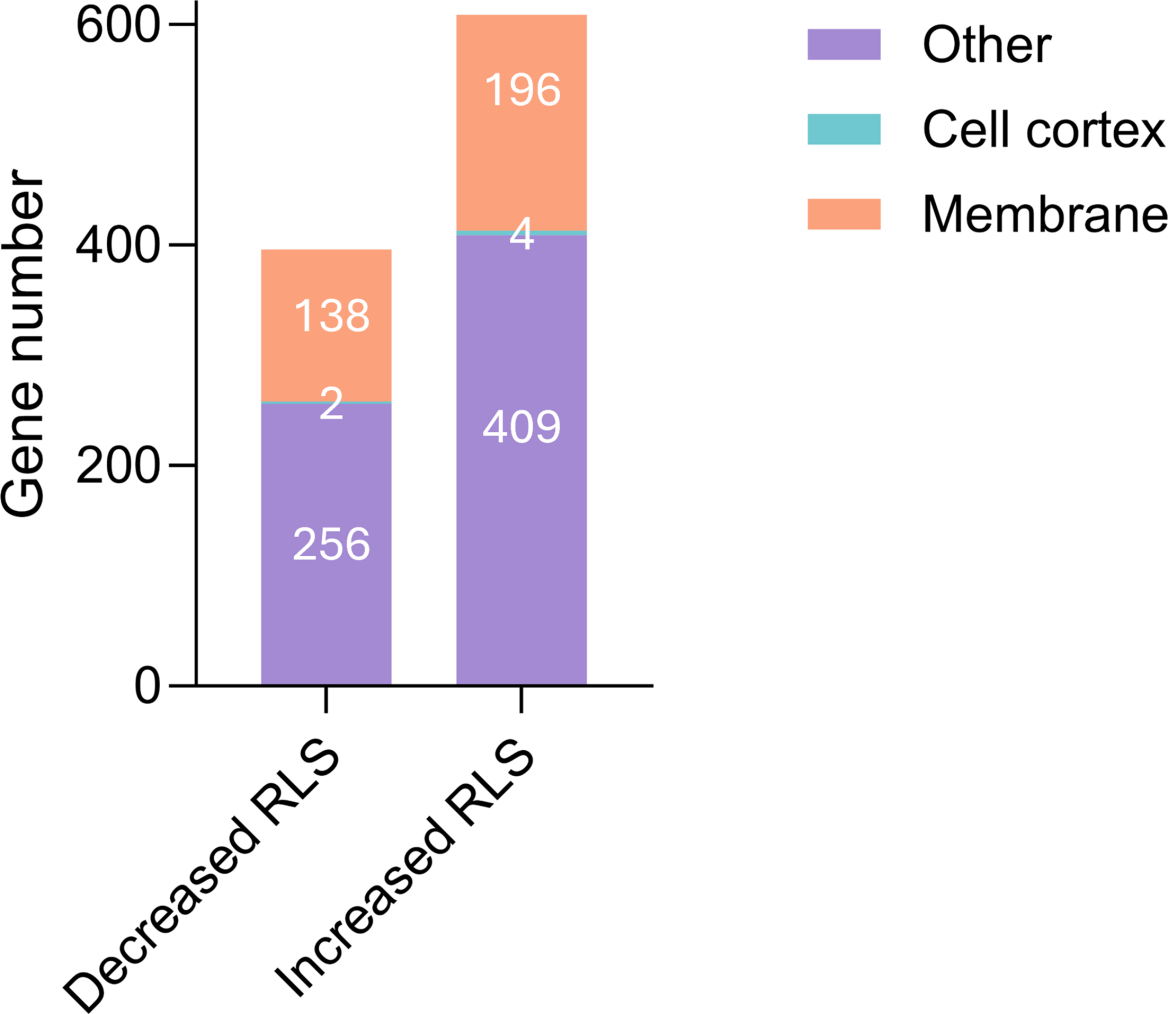
Distribution of membrane and cortex-localized protein-encoding genes. Based on experimental evidence curated in the SGD, deletion of 396 genes decreases RLS, and deletion of 609 genes increases RLS in the *S. cerevisiae* S288C reference strain. For genes with conflicting reports, the RLS phenotype supported by most publications was considered. Protein localization was assigned using gene ontology (GO) annotations for ‘membrane’ (GO:0016020) and ‘cellular cortex’ (GO:0005938). The ‘membrane’ category includes proteins embedded in or attached to lipid bilayers, while the ‘cellular cortex’ refers to proteins associated with the actin-rich layer beneath the plasma membrane. Six genes annotated with both terms were included in the ‘membrane’ category. GO annotations were obtained from computational analyses included in SGD.

Several genome-wide analyses have determined the RLS phenotype of *S. cerevisiae* single-gene deletion mutants [30,67–69]. An examination of these reports and the *Saccharomyces* Genome Database (SGD) indicates that approximately 30% of the mutants correspond to genes encoding proteins localized at the cell membranes or the cell cortex (Figure 1).

Here we describe five research works that have investigated protein asymmetric segregation using a variety of methodological approaches. First, Yang et al. [5] reported 74 asymmetrically distributed, mother-enriched proteins. Cells from the green fluorescent protein (GFP) tagging library were pulse-labeled with the fluorescent dye Cy5 to mark mother cells, enabling their distinction from unlabeled daughter cells and subsequent GFP signal monitoring by flow cytometry.

Second, Okada et al. [7] identified 21 proteins asymmetrically inherited in mother cells. In a synchronized culture, newly synthesized proteins were labeled with stable isotope amino acids to discriminate against pre-existing proteins originally present in mother cells. Separation of mother and daughter cells was achieved by biotin labeling followed by streptavidin binding. Isotope incorporation ratios for individual proteins were quantified by mass spectrometry. In this study, 5 of the 21 identified proteins (Zrt1, Thr1, Itr1, Pet10, and Pdr5) were previously detected by Yang et al. [5].

Third, in Sugiyama et al. [8], G1-arrested cells were stained with Cy5, released from the cell cycle arrest, and then newly synthesized proteins were labeled by pulsed stable isotope labeling in cell culture (pSILAC). Cell sorting followed by mass spectrometry analysis determined the ratios of pre-existing to newly synthesized proteins in both daughter and mother cell fractions. This work identified 56 mother cell-enriched proteins, with 35 of these not detected in the other two screens described.

Fourth, Thayer et al. [9] analyzed asymmetry by comparing mother cells that had undergone, on average, 18 cell divisions with their resulting daughter cells. Using a combination of pSILAC with a biotin-streptavidin-based strategy to enrich the cell culture with aged cells, the authors identified 21 asymmetrically segregated, long-lived proteins and confirmed asymmetric distribution of five of them (Snq2, Mrh1, Pma1, Bgl2, and Thr1) by fluorescence microscopy.

Finally, Eldekak et al. [6] identified a group of 14 plasma membrane proteins belonging to the multidrug resistance protein families [70] by analyzing the GFP-tagged budding yeast protein-localization database [71]. The distribution of these proteins was strongly biased towards the mother before the onset of anaphase. Five of these proteins (Fui1, Hnm1, Hip1, Vht1, and Top1) were not detected in the other screens.

**Figure 2:**
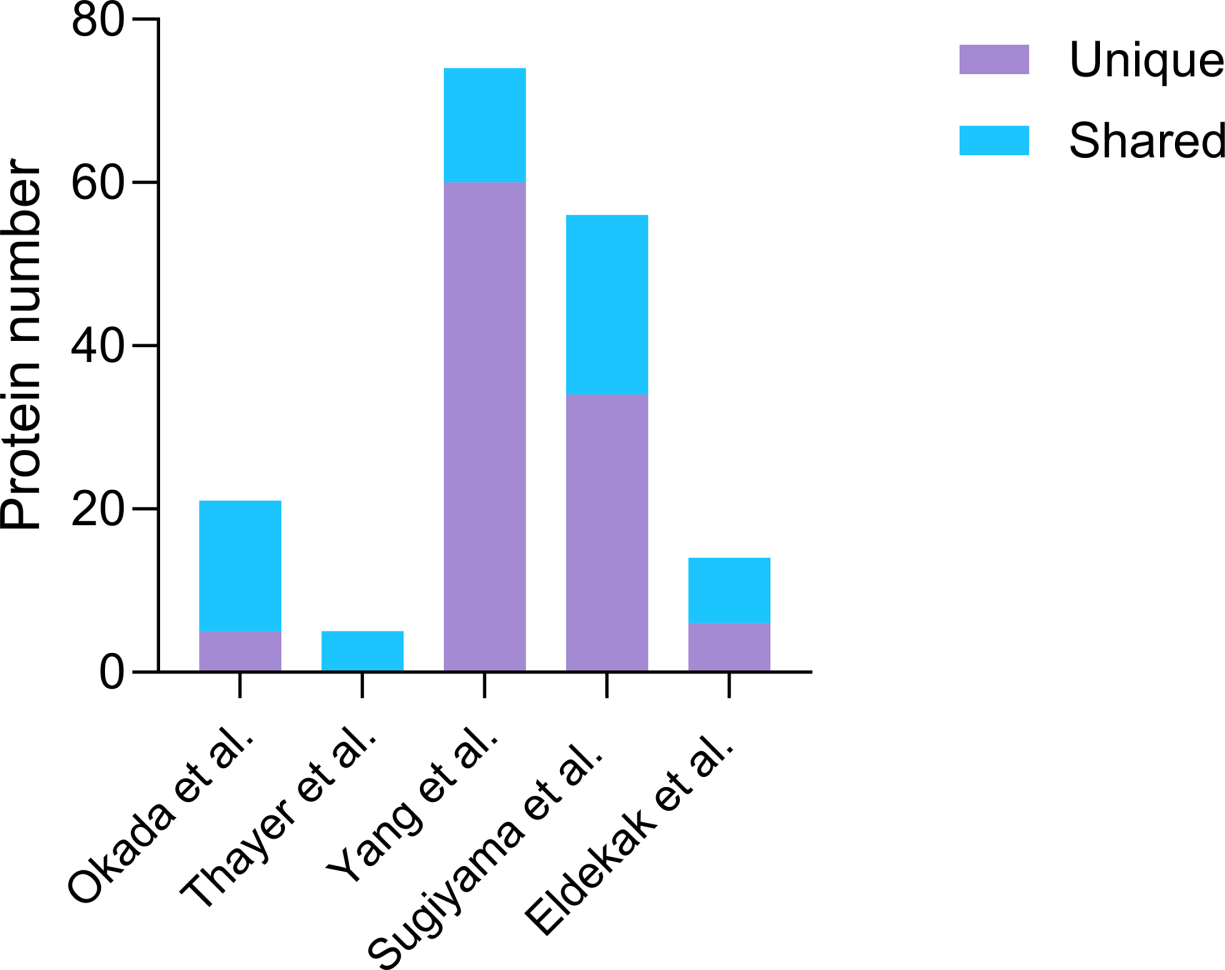
Mother-enriched proteins identified across five proteome-wide studies. Mother-enriched proteins are those that preferentially accumulate in mother cells during asymmetric cell division in budding yeast. The haploid *S. cerevisiae* genome encodes ~6600 protein-coding genes, and subsets of these were identified as mother-enriched in five independent large-scale proteomic screenings [5,7–9] and [6]. The number of proteins identified is shown. Proteins uniquely detected in a single study are shown in violet, whereas proteins identified in at least two studies are indicated in cyan.

Analysis of the different sets identified by these methods reveals a total of 134 proteins that are enriched in the mother cell. The majority were uniquely identified by a single approach (Figure 2), highlighting the importance of employing diverse methodologies for the comprehensive identification of asymmetrically segregated proteins. Supplementary Table S1 details the intersections among the five compiled datasets. In addition, Yang et al. [5] report a set of daughter-enriched proteins adding 58 proteins to the analysis (Supplementary Table S1).

### 3.2 Shared Proteins between Asymmetric Segregation and Lifespan Modulation

**Figure 3:**
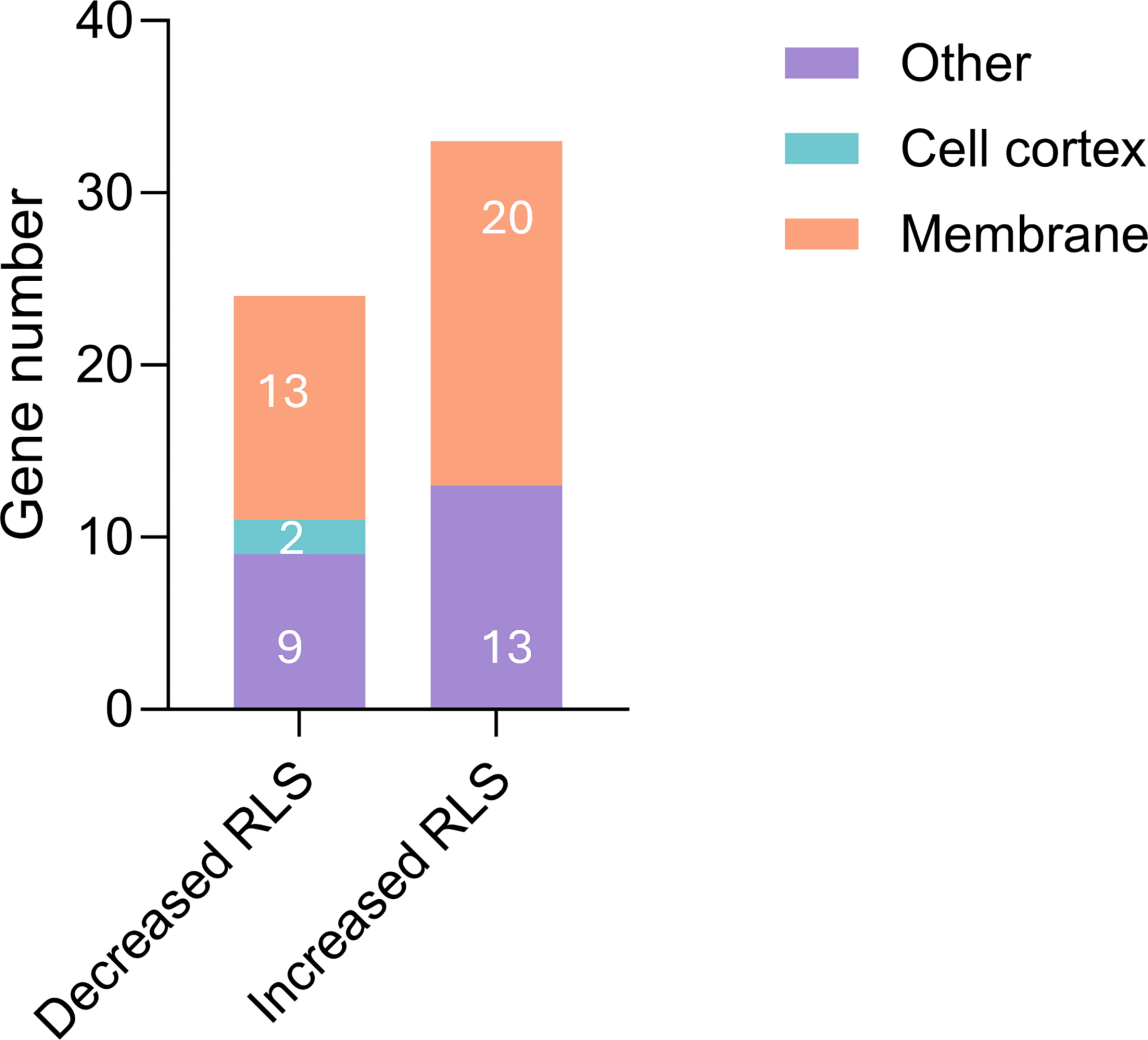
Number of genes codifying for asymmetrically segregated proteins. Based on experimental evidence curated in SGD, 57 null mutants in genes encoding asymmetrically segregated proteins exhibit a significant change in RLS. References for the experimental evidence for each gene are listed in Supplementary Table S1. Protein localization was assigned using GO cellular component annotations, listed in Supplementary Table S1.

An examination of the integrated set of 192 asymmetrically segregated proteins shows that 57 deletion mutants have statistically different RLS from wt strain (Supplementary Table S1). Interestingly, the fraction of long-lived gene deletions (33 out of 192, 17.2%) is 3.5 times the frequency of lifespan extension observed in viable genome-wide gene deletions (228 out of 4698, 4.9%, [67]). Over 60% (35 out of 57) of asymmetrically segregated proteins that affect RLS are membrane- or cell cortex-associated (Figure 3), almost twice the proportion of that calculated for the complete set of lifespan deletion mutants (340 out of 1005, 33.8 %).

**Figure 4:**
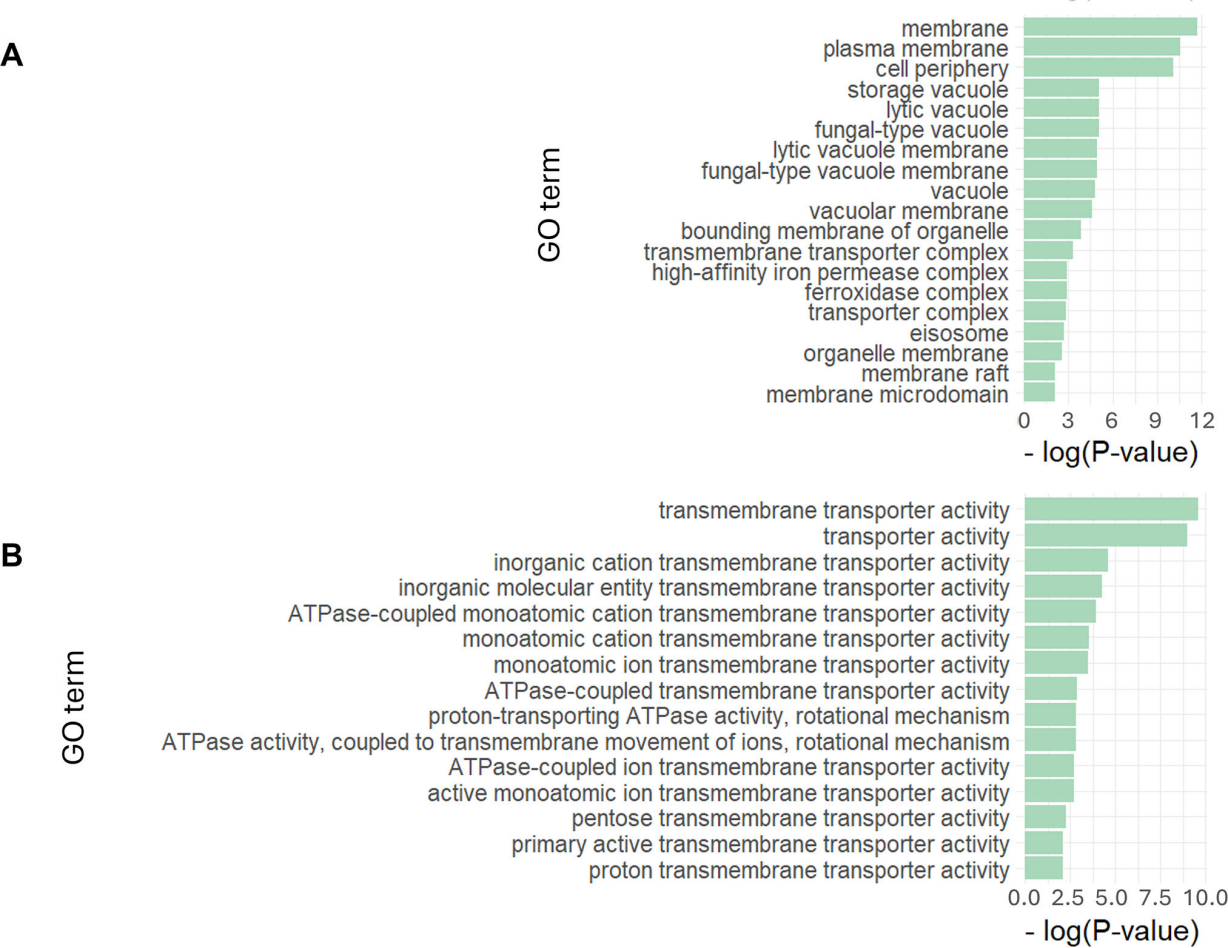
GO analysis of genes codifying for asymmetric membrane proteins involved in RLS. **A**.GO enriched categories, compared to whole yeast genome analysis (*P*<0.01), associated with a specific cellular component are shown. Note that the plasma membrane is a subcategory of the broader ‘membrane’ GO term (*P=*1.3 10^–16^) and cell periphery includes the plasma membrane, cell cortex (inside the cell), and any external structures (cell wall). **B**. Significant GO categories associated with a specific function. Analysis was performed with the GO term finder of SGD.

The analysis enabled the identification of a group of 35 asymmetrically segregated proteins localized at cellular membranes or at the cell cortex whose deletion mutants show altered RLS (Supplementary Table S1). Gene ontology enrichment analysis confirms that genes encoding membrane proteins are significantly overrepresented (Figure 4A). Moreover, the analysis identified proteins localized to intracellular membranes, including the nuclear, mitochondrial, and vacuolar membranes, as well as the plasma membrane and cellular cortex. A description for each gene is detailed in Supplementary Table S2.

This set of asymmetrically distributed membrane proteins involved in aging includes proteins detected in high-throughput RLS analyses, as well as proteins already well-described in the literature. Genes encoding proteins involved in transmembrane transport activity are overrepresented (Figure 4B). Deletion of three genes (*MEP2, TPO3,* and *CAN1*) that encode nitrogen compound plasma membrane transporters extends lifespan [67]. While the mechanism underlying Mep2 and Tpo3 pro-aging roles is unknown, Beaupere et al. [72] show that a null mutant for the Can1 arginine permease exhibits an extended RLS that depends on activation of the transcription factors Gcn4 and Hac1. Mep2, Tpo3, and Can1 asymmetric segregation is consistent with previous reports showing that many transporters in the plasma membrane are retained in mother cells due to their slow diffusion [6,73]. However, future research is needed to elucidate the mechanisms underlying the asymmetric segregation of these proteins. This work should include protein diffusion measurements and determining the role of the septin-based barrier in restricting their movement [74]. In addition, it remains to be determined whether the loss of asymmetry of these transporters impacts RLS.

As described in section 2.4, Pma1, which accumulates as mother cells age, antagonizes vacuolar acidification [7–9,60]. Consistent with these results, cells with less Pma1 activity live longer [9,60]. Recent research has implicated components of the endocytic pathway in regulating Pma1 asymmetry. Mutations in vacuolar protein sorting (VPS) genes such as *VPS8*, *VPS9*, and *VPS21*, which are involved in endocytosis, can disrupt the asymmetric distribution of Pma1 [66]. Moreover, Yoon et al. [66] found that the RLS of yeast cells deficient for *VPS9* or *VPS21* was not significantly different from that of wildtype cells, suggesting that loss of Pma1 mother enrichment, per se, is not sufficient to increase RLS. It remains to elucidate if in these mutants, Pma1-enriched daughter cells are less acidic [60]. Also, it would be insightful to determine whether deleting *VPS* genes affects the distribution of other asymmetrically localized membrane proteins that influence RLS, and whether this loss of asymmetry contributes to the RLS phenotypes of VPS deletion mutants.

Several asymmetrically segregated vacuolar proteins have been identified. Vma5, a V1 subunit that bridges the V1 and V0 subcomplexes of the V-ATPase is enriched in mother cells [5]. Recently, it was demonstrated that V-ATPase disassembles in aging cells with release of Vma5 into the cytosol [75]. V-ATPase disassembly is observed after 5 cell divisions and correlates with an overall vacuole alkalinization. Hashmi et al. propose that this process further contributes to the loss of vacuolar acidification with age, which reduces lifespan. Authors found that reversible disassembly is controlled in part by the activity of two opposing and conserved factors: the regulator of acidification of vacuoles and endosomes (RAVE) complex and Oxr1. The RAVE complex promotes V-ATPase assembly, whereas Oxr1 promotes disassembly. Loss of Rav1, a subunit of the RAVE complex, shortens RLS while overexpression of another subunit, Rav2, delays V-ATPase disassembly with age. Conversely, *OXR1* gene deletion extends RLS [75]. It is unknown if canonical aging pathways are involved in the regulation of V-ATPase reversible disassembly and to what extent this process depends on Pma1 activity at the plasma membrane.

Another vacuolar membrane protein, Vac8, is accumulated in mother cells, and its gene deletion leads to a decrease in RLS [68,76]. A genome-wide screen revealed *VAC17* and *VAC8* as genes required for generating asymmetric inheritance of protein aggregates [45,77]. Hill et al. suggest that *VAC8* could play a role in the retention of protein aggregates in aged mother cells [45]. Overexpression of *VAC17* promotes the formation of vacuole-proximal PI in the mother cell and protein aggregates asymmetry. Moreover, this improved asymmetry is associated with an extended RLS [1,45].

Finally, mitochondrial membrane proteins that are enriched in mother cells were also identified, including the cytochrome C protein Cyc1, pyruvate dehydrogenase protein Pda1, and the ATPase components Atp1 and Atp2. Mutations in the *ATP2* gene, encoding the β subunit of the F1 subunit of the mitochondrial ATP synthase, lead to loss of mitochondrial membrane potential, accumulation of dysfunctional mitochondria, decrease in the number of active segregated mitochondria, and shorter RLS [78].

## 4. Future directions

This review provides a comprehensive resource of asymmetrically distributed membrane proteins in budding yeast. Several interesting questions remain concerning asymmetry establishment and maintenance and their precise impact on lifespan control.

Fitter mitochondrial asymmetric segregation to the daughter cell is largely established. The cytoskeletal role in the movement of these organelles to and from the bud has been extensively studied as well. However, future research will be needed to understand the mechanisms underlying mitochondrial quality control pathways. Crucially, identifying the specific signals and protein machineries that link mitochondrial fitness detection with plasma membrane tethering and asymmetric sorting remains an important area of investigation.

The precise role of ERCs as aging promoters remains to be fully addressed. The mechanism governing the asymmetric segregation of NPC-anchored ERCs is largely unexplored. The extent to which the natural geometry of budding yeast cell division contributes to ERC asymmetric distribution also remains to be determined.

While significant progress has been made in understanding asymmetric protein-aggregate segregation, several fundamental questions remain unanswered: are there specific post-translational modifications of PQC components that regulate their activity and contribute to their age-related decline? Which are the specific organelle components that capture and retain protein aggregates in mother cells, and to what extent do diffusion barriers facilitate this segregation? Beyond the established PIs (JUNQ, IPOD, and INQ), it would be interesting to identify whether other cellular sites or novel mechanisms are involved in the sequestration of protein aggregates, specifically within aging cells. Finally, research is needed to understand how anti-aging factors like Sir2 regulate actin assembly and contribute to asymmetric aggregate partition and to identify the upstream signals that influence Sir2 activity in this context.

While vacuolar acidification is a recognized key factor in aging, many questions surrounding this process are still open. Future research will be needed to clarify whether Pma1’s asymmetric localization influences RLS. Although significant progress has been made describing the mechanism of reversible assembly of the V-ATPase, the specific age-associated signals that regulate this process still require elucidation. Of particular interest, the signal triggering V-ATPase disassembly in aged cells remains unidentified.

Given that more than 30% of the membrane proteins described as asymmetrically delivered to the membrane are involved in RLS, future studies analyzing their role in aging offer a promising approach to understanding the mechanisms of regulated asymmetric aging.

## Supplementary material

online supplementary material 1

Online supplementary table 1

Online supplementary table 2

## Open Access

This article has been published open access under our Subscribe to Open programme, made possible through the support of our subscribing institutions, learn more here: https://portlandpress.com/pages/open_access_options_and_prices#conditional

## Abbreviations

CN: copy number
ERCs: extrachromosomal rDNA circles
GFP: green fluorescent protein
GO: gene ontology
INQ: intranuclear quality control site
IPOD: insoluble protein deposit
JUNQ: juxtanuclear quality control site
MECA: mitochondria-ER-cortex-anchor
NPCs: nuclear pore complexes
PIs: protein inclusions
PQC: protein quality control
RAVE: regulator of acidification of vacuoles and endosomes
RLS: replicative lifespan
ROS: reactive oxygen species
SGD: *Saccharomyces* Genome Database
SLCs: stem-like cells
V-ATPase: vacuolar proton-ATPase
VPS: vacuolar protein sorting
pSILAC: pulsed stable isotope labeling in cell culture
