# Supplementary material for: Cell membrane asymmetries and cellular aging
Source: Biochem J. 2025 Oct 9;482(20):1517–29. doi: 10.1042/BCJ20253265 (PMC12687462; doi:10.1042/BCJ20253265)
Supplement: online supplementary material 1 [file bcj-482-20-BCJ20253265-s001.docx]

**Supplementary material**

**Supplementary table 1. Asymmetrically segregated proteins identified across five proteome studies.**

The table includes 11 columns (A-K) with (A) protein name; (B) systematic gene name; presence (X) in proteome studies is indicated for asymmetric segregated proteins in (C) Okada [et al](http://et.al). [[7]](https://paperpile.com/c/Zf04vD/0MGOr), (D) Thayer et al. [[9]](https://paperpile.com/c/Zf04vD/h328), (E) Yang et al. [[5]](https://paperpile.com/c/Zf04vD/ZnF3S), (F) Sugiyama et al. [[8]](https://paperpile.com/c/Zf04vD/oNVTc) and (G) Eldekak et al. [[6]](https://paperpile.com/c/Zf04vD/FdCaV)**;** (H) asymmetry segregation in the mother or daughter cells is indicated as “mother”, “daughter” or “mother or daughter”; (I) GO term assignment; (J) RLS null mutant phenotype (decrease or increase qualifiers); (K) PMID related to RLS phenotype determiantion.

Notes in reference to columns I, J and K of Supplementary table 1:

(I) Genes annotated with the "membrane" GO term (GO:0016020) in computationally derived SGD data, or with child terms containing "membrane" in manually curated SGD data, are designated as “membrane”. Genes annotated with the "cell cortex" GO term (GO:0016020) in computationally derived SGD data, or with child terms containing "cell cortex" in manually curated SGD data, are designated as “cell cortex”.

(J) SGD database information for null S288C strains except for PMA1 RLS phenotype that was determined in a mutant with reduction of function (Allele: [pma1-105](https://www.yeastgenome.org/allele/pma1-105)). Where different analyses yielded opposing results, the phenotype with the greater number of reports was selected.

(K) PMID related to RLS phenotype. Only one PMID associated with each gene is shown.

**Supplementary table 2. Asymmetric segregated membrane proteins that modulates RLS**

The table includes 3 columns (A-C): (A) gene, (B) systematic gene name and (C) gene description obtained from SGD.
